# Ventricular Septal Rupture Following Acute Myocardial Infarction

**DOI:** 10.7759/cureus.29848

**Published:** 2022-10-02

**Authors:** Yachika Thakkar, Jay Thakkar, Sourya Acharya, Samarth Shukla, Sandeep Kamat, Tarun Rao, Sunil Kumar

**Affiliations:** 1 Department of Medicine, Jawaharlal Nehru Medical College, Datta Meghe Institute of Medical Sciences, Wardha, IND; 2 Department of Pathology, Jawaharlal Nehru Medical College, Datta Meghe Institute of Medical Sciences, Wardha, IND; 3 Department of Cardiology, Topiwala National Medical College and Bai Yamunabai Laxman (BYL) Nair Charitable Hospital, Mumbai, IND

**Keywords:** myocardial infarction, st-elevation myocardial infarction (stemi), electrocardiography, doppler echo, ventricular septal rupture

## Abstract

ST-segment elevation myocardial infarction (STEMI) is a known medical exigency that has seen considerable advances in medical treatment, dramatically boosting survival rates. Post myocardial infarction ventricular rupture is a major serious mechanical complication following myocardial infarction. We present a case of a 68-year-old male admitted to the emergency department with heaviness in the chest, for which electrocardiography was done and it was suggestive of anterior and lateral wall myocardial infarction. After six hours he experienced breathlessness, jugular venous pressure (JVP) was raised, and auscultation revealed early systolic murmur at apex suggestive of ventricular septal rupture. An urgent echocardiogram was done and it confirmed the diagnosis of ventricular septal rupture (VSR). To enhance the prognosis, early identification and appropriate care are required, which necessitate a thorough clinical evaluation that raises the possibility of mechanical problem, as late presentation is one of the major risk factors for developing VSR. VSR can manifest itself in numerous ways, based on the patient’s condition. Right clinical judgement and ECG are required to establish a quick diagnosis, as a result, to determine the most appropriate treatment at the appropriate time.

## Introduction

Acute myocardial infarction (AMI) is a major cause of death in the world, affecting almost three million people globally and resulting in over one million deaths in the United States each year. Because of the absence of oxygen, myocardial infarction (MI) causes unrecoverable damage to cardiac muscles [1-3]. MI in the anterior chamber is more common (60%) than in the inferior chamber (40%). Ventricular septal rupture (VSR) takes place in about 1% of sufferers who have had an acute MI, making it a relatively uncommon mechanical consequence [4,5]. Clinical examination, electrocardiography, colour Doppler echocardiography, and left cardiac catheterization are used to diagnose and evaluate the condition [6].VSR is a deadly complication of AMI. Old Age, anterior infarction, and a history of smoking are factors associated with VSR worsening AMI. Incomplete revascularization and cardiogenic shock at the time of operation were also revealed to be strong absolute determinants of bad 30-day and lifelong survival [7,8]. The mechanical consequence usually occurs 10-14 days post-MI, when necrotic tissue is common and collateral coronary circulation is still poor [9]. VSR has a two-month mortality rate of 87% in individuals treated with medicines [10], implying that VSR is lethal unless it is repaired through surgery. According to certain statistics, one-third of patients die before they reach the hospital, and 40% to 50% die there. Readmission occurs in 50% of patients. Patients who do not have any revascularization will have a worse outcome than those who do [11-13]. 

## Case presentation

**Figure 1:**
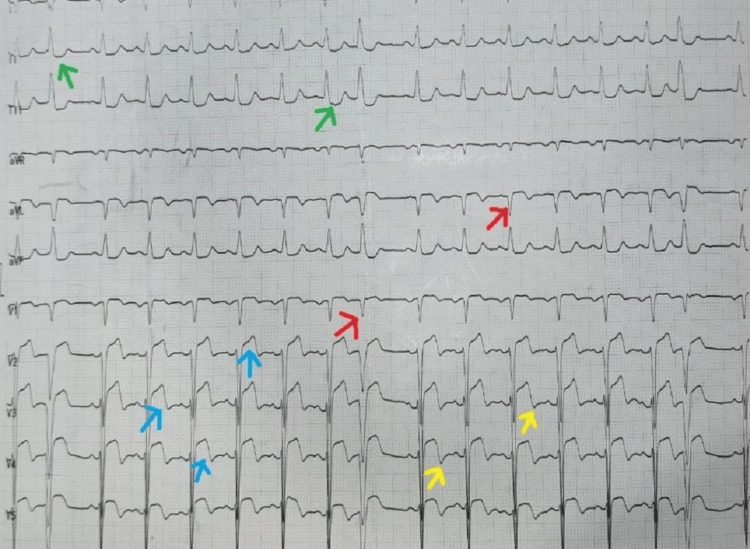
12 lead ECG showing Q waves (arrows in red colour) in leads 1, aVL, and V1-V6, ST-segment elevation (arrows in blue colour) in leads 1, aVL, and V1-V6, and T-wave inversions (arrows in yellow colour) in leads 1, aVL, and V1-V6, with reciprocal ST-segment depression (arrows in green colour) in inferior leads suggestive of anterolateral wall myocardial infarction

**Figure 2:**
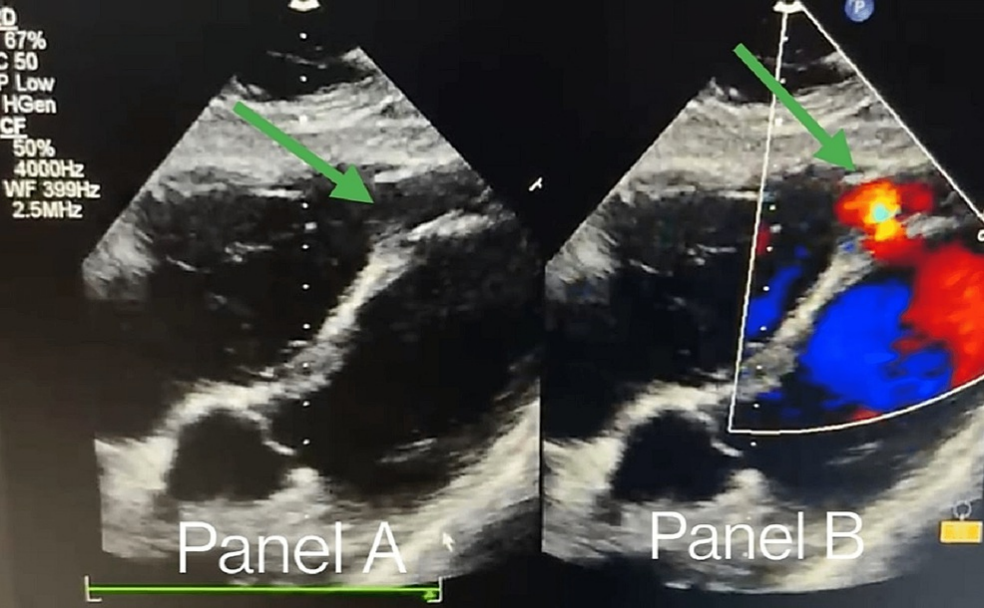
Panel-A: Sub-costal view showing apical muscular ventricular septal rupture (green arrow) following myocardial infarction. Panel-B: Sub-costal view showing left to right shunt across apical muscular ventricular septal rupture (green arrow) following myocardial infarction

A 68-year-old male presented to the emergency department of this hospital in the morning at 8:30 am with a chief complain of chest pain radiating to his left arm and jaw with a history of chest discomfort, post-dinner on the previous night. He took an antacid and went to bed, considering it was due to gastritis. He had experienced a similar ache in the middle of the night associated with diaphoresis which lasted for 10-12 minutes, took the same medication, and went to sleep. In the morning when he got up he still had heaviness in the chest for which he presented to the hospital. There was no history of breathlessness, pre-syncope, cough, orthopnea, or vomiting. Clinical examination revealed pulse to be 100 beats per min and regular blood pressure of 110/70 mm Hg. JVP was normal. Oxygen saturation was 95% while breathing ambient air. A cardiovascular (CVS) examination revealed a soft S1, normal S2, no S3, and no murmurs. Respiratory system examination revealed minimal bi-basal scattered crept. The rest of the examination was unremarkable. An urgent ECG revealed an elevated ST segment in the anterior and lateral leads. ECG was suggestive of anterolateral wall MI (Figure 1). Laboratory findings revealed the following: Hb-12 gm%, TLC-11000/cubic mm, creatine phosphokinase-myocardial band-115 IU/L, kidney function test (KFT) and liver function test (LFT) were normal, and cardiac markers revealed cardiac-specific troponin I-0.89 ng/ml (Table 1). The patient was started on a dual antiplatelet, high-dose statin, injected low molecular weight heparin, Beta-blockers, and diuretics. Coronary angiography was planned for which the patient's relatives did not give consent. Six hours after admission he again started complaining of breathlessness. Examination revealed tachycardia, heart rate-130/min, blood pressure-80 mm Hg systolic, JVP was raised, and auscultation revealed grade 2/6 early systolic murmur at the apex. Clinically VSR was suspected and urgent echocardiography revealed acute VSD (Figure 2).

**Table 1:** Lab diagnosis gm% = gram percent; mg/dl = milligram per deciliter; U/L = units per liter; g/dl= gram per deciliter; IU/L= international units per liter; ng/ml = nanogram per milliliter; cu.mm = per cubic millimeter; m Eq/L= milliequivalents per liter; AST = serum aspartate transaminase; ALT = serum alanine transaminase; CPK-MB = creatine phosphokinase-myocardial band

Test Name	Result	Normal range
Haemoglobin%	12	Male: 12.0-16.0 gm% Female: 11.0-14.0 gm%
Total Leucocyte Count	11000	4000-11000 / cu.mm
Creatinine	0.9	Male: 0.7-1.3 mg/dl Female: 0.6-1.1 mg/dl
Urea	14	Male: 8-24 mg/dl Female: 6-21 mg/dl
Sodium	138	135-145 mEq/L
Potassium	3.8	3.5-5.0 mEq/L
AST	22	Male: 10-40 U/L Female: 9-32 U/L
ALT	19	Male: 7-35 U/L Female: 7-40 U/L
Total Bilirubin	0.7	0.1-1.2 mg/dl
Serum Protein	6.8	6-8 g/dl
CPK-MB	115 IU/L	0-16 IU/L
Troponin- I	0.89 ng/ml	0-0.04 ng/ml

The patient was immediately started on inotropic support and put on intra-aortic balloon pump (IABP); urgent cardiothoracic surgery consultation was taken but the patient succumbed within the next hour. 

## Discussion

In the current era of percutaneous coronary intervention, ventricular septal rupture following an acute myocardial infarction is not common, although mortality is still high. The importance of early detection cannot be overstated, and definitive surgery, despite its difficulty and high mortality rate, remains the preferred therapeutic option. In both anterior and inferior/lateral infarctions, ventricular septal rupture appears to be equally common [14]. Female gender, elderliness, hypertension, anterior wall MI, lack of previous MI, and delayed arrival are all linked to a greater risk of mortality, according to Serpytis et al. Several studies have discovered that the time between the onset of symptoms and the surgery is the most important risk factor for operating mortality and intra-hospital survival, with prompt surgical intervention being associated with a higher death rate [15-17]. VSR is common as a simple defect in anterior wall AMIs compared to complicated VSR in inferior AMIs. Inferior AMIs are more likely to have numerous channels connecting the left and right ventricles. The link between the two ventricles causes a pathologic left to right shunt, which appears as right ventricular volume overload; as a result, cardiac output is reduced; bundle branch blockage is frequently caused by worsening of septal conduction which can result in cardiogenic shock [18]. The majority of VSRs occur three to five days after AMIs [19].

ECG is crucial for determining the location of a VSR. It indicates that in many of the individuals having VSR, like in our instance, the anterior part is more commonly afflicted (60%) rather than the inferior area (40%) [20]. It is critical to employ echocardiography to diagnose VSR. It allows for the quick diagnosis of the shunt, the location, and the rupture extent, along with the avoidance of mechanical difficulties. VSR patients must be treated as soon as possible. To minimize the left-right shunt, medical therapy focuses on lowering afterload with an intravenous vasodilator and an intra-aortic balloon pump. Because individuals who are treated purely medically have a dismal prognosis, with in-hospital death rates ranging from 94% to 100%, this therapy is just a temporary option. Among those who have experienced an acute MI, patients with post-infarction ventricular septal defect represent a highly high-risk subset. The related hemodynamic instability, complex nature of the defect, and its progression over time as infarcted myocardium is resorbed are significant clinical and anatomical problems. Although both transcatheter and surgical VSD closure have substantial mortality rates, they are both significantly lower than medical therapy alone [21-24]. Despite its high morbidity and mortality, surgery is the preferred therapy, whatever be the hemodynamic stability during the time of diagnosis. The American College of Cardiology Foundation and the American Heart Association recommended rapid VSR repair following an acute MI [25].

## Conclusions

Despite recent advancements, post-MI VSD is a fatal consequence. The gold standard is early clinical diagnosis and immediate surgical intervention. Pre- and post-operative care provided, in addition to surgical treatment, is critical for improving survival rates.
